# Comparative Analysis of Recent Burn Guidelines Regarding Specific Aspects of Anesthesia and Intensive Care

**DOI:** 10.3390/ebj6040057

**Published:** 2025-10-29

**Authors:** Rolf K. Gigengack, Joeri Slob, J. Seppe H. A. Koopman, Cornelis H. Van der Vlies, Stephan A. Loer

**Affiliations:** 1 Department of Trauma and Burn Surgery, Maasstad Hospital, Maasstadweg 21, 3079 DZ Rotterdam, The Netherlands; 2 Department of Intensive Care, Amsterdam UMC, De Boelelaan 1117, 1081 HV Amsterdam, The Netherlands; 3 Department of Anesthesiology, Amsterdam UMC, De Boelelaan 1117, 1081 HV Amsterdam, The Netherlands; 4 Alliance of Dutch Burn Care (ADBC), Burn Center, Maasstad Hospital, Maasstadweg 21, 3079 DZ Rotterdam, The Netherlands; slobj@maasstadziekenhuis.nl; 5 Department of Anesthesiology, Maasstad Hospital, Maasstadweg 21, 3079 DZ Rotterdam, The Netherlands; 6 Trauma Research Unit Department of Surgery, Erasmus MC, University Medical Centre Rotterdam, Dr. Molewaterplein 40, 3015 GD Rotterdam, The Netherlands

**Keywords:** burn guidelines, airway management, mechanical ventilation, burn resuscitation, pain management, procedural sedation management

## Abstract

Background: Critical care for patients with severe burn injuries is challenging, particularly in the first 24–48 h. Multiple guidelines exist but their recommendations vary in content and in the level of detail. Methods: This narrative review analyzed recent (last 10 years) adult burn guidelines in English, Dutch and German, sourced from PubMed, Medline and official burn society publications. The review focused on airway management, mechanical ventilation, fluid resuscitation, pain management and procedural sedation. Results: All guidelines emphasize early airway assessment and timely intubation in patients at risk for loss of airway patency; however, a strategy for analyzing patients at risk is lacking. Lung-protective ventilation strategy is generally recommended. Fluid resuscitation is the cornerstone during the first phase, though recommendations for thresholds, volume and adjuncts differ. (Chronic) pain management should be multimodal, combining pharmacologic and non-pharmacologic approaches, but specifics on choice of modality are limited, also, there is no uniform strategy for procedural sedation management. Conclusion: Current guidelines offer broadly consistent recommendations for initial burn care but differ in specifics, reflecting evidence gaps. Future guidelines should address advances in airway management, fluid resuscitation endpoints, volume and adjuncts, and give a more detailed (chronic) pain strategy to improve standardization and outcomes.

## 1. Introduction

Critical care of patients with severe burn injury (>15% TBSA) remains challenging and requires special knowledge and experience. In the initial phase (the first 24 to 48 h), maintaining vital functions such as respiration and circulation is crucial. Accordingly, various aspects of airway management, mechanical ventilation, fluid resuscitation and pain treatment play a prominent role in early management [1,2].

Several national and international burn guidelines, including the guidelines of the European Burn Association (EBA), the American Burn Association (ABA), the British Burn Association (BBA), the International Society for Burn Injuries (ISBI) and the German Society for Burn Medicine (DGV) provide recommendations for the management of burn patients [3,4,5,6,7,8,9]. As expected, these guidelines overlap in most recommendations, while differing in others. Recently, Koyro et al. described the similarities and differences between the latest recommendations of six well-known burn societies and analyzed the (dis)advantages of each guideline. They focused on structure, readability, transfer criteria and overall content without comparing the content in detail [10]. Similarly, Paprottka et al. compared the German, European, and American burn guidelines at a more meta-level, comparing readability, completeness, timeliness and overall content without focusing on specific differences in clinical management [11]. To date, no in-depth review has examined the recommendations of the current guidelines for the management of vital functions of adult burn patients in the first 24–48 h. Therefore, this narrative review compares how different guidelines address specific anesthesiological and intensive care aspects during the initial treatment period. Our focus is on airway management and mechanical ventilation, fluid resuscitation, pain management and procedural sedation.

## 2. Materials and Methods

In our narrative review, we considered recent burn treatment guidelines (published within the past 10 years) in English, Dutch or German found through literature searches (PubMed, Medline) or were publicly available from national or international burn societies. We limited our analysis to guidelines that provided recommendations for adult burn patients. Recommendations with respect to the management of vital functions during the initial resuscitation phase of treatment (24–48 h) were extracted. Specifically, recommendations on airway management and mechanical ventilation, fluid resuscitation, pain management and procedural sedation.

## 3. Results

The guidelines of the European Burn Association (EBA) [4], the American Burn Association (ABA) [12], the German Society for Burn Medicine (DGV) [8], the British Burn Association (BBA) [7], the International Society for Burn Injuries (ISBI) [3,9], Japanese Society for Burn Injuries (JSBI) [13], Irish Association of Emergency Medicine (IAEM) [14], Eastern Association for the Surgery of Trauma (EAST) [15], and the Medicines sans Frontieres (MSF) [16] were included in this comparative analysis.

### 3.1. Airway Management and Mechanical Ventilation

A key issue in the acute care of burn trauma patients remains airway management, particularly in patients at risk of airway obstruction following inhalation trauma or direct thermal injury. Table A1 summarizes the recommendations on airway management and Table A2 the recommendations on mechanical ventilation. In this early phase, clinicians must continuously assess and reassess airway patency at adequate intervals and timely secure the airway with an endotracheal tube if patency is at risk (DGV and ISBI recommendations) [8,9]. The decision to intubate is influenced by multiple factors, making it complex and challenging. Endotracheal intubation is an invasive procedure associated with various risks; on the other hand, an endotracheal intubation secures airway patency and eliminates the risk of airway compromise (recommendations of DGV, JSBI and ISBI) [8,9,13]. Scoring systems developed to support this decision still lack sensitivity and specificity. A higher sensitivity often comes at the expense of specificity [17]. The ISBI, DGV, IAEM and EAST guidelines contain specific recommendations for the decision to intubate [8,9,14,15]. They highlight the risk of loss of airway patency due to swelling and mucosal injury from thermal damage. Common indication for intubation includes acute respiratory failure, loss of protective reflexes, airway obstruction, inspiratory stridor and severe cognitive impairment [8,9,14,15]. Burn-specific indication includes systemic inhalation injury (e.g., carbon monoxide poisoning), extensive burns (>40% TBSA) and signs of burns to the oropharynx (e.g., blistering of the mucosa, hoarseness and stridor development) [8,9,14,15]. Careful and continuous airway monitoring is crucial when considering and deciding for deferred intubation [8,9,13,15]. At the same time, when decided to intubate, the physician should assume that burn patients will have a difficult airway. Clinicians must be prepared accordingly and follow the airway management guidelines for difficult airways [18]. The ISBI guideline specifically recommends that the most experienced physician performs the procedure to intubate [9,14].

With respect to mechanical ventilation in burn patients, two guidelines (DGV and ISBI) recommend the use of lung-protective ventilation strategy. The DGV guideline recommends the use of a PEEP-value of ≥5 cm H_2_O, tidal volumes of 6–8 mL/kg, a maximum plateau pressure of 30 cm H_2_O and a maximum driving pressure of 15 cm H_2_O. [8] In contrast, the ISBI guideline provides a more general description of lung-protective ventilation strategy and recommends the use of the lowest possible ventilation pressures and tidal volumes without specifying specific volumes or pressures [9]. To achieve lung-protective ventilation in cases of respiratory failure, the DGV guideline further recommends using permissive hypercapnia and prone positioning and considering veno-venous extracorporeal membrane oxygenation (vv-ECMO) as rescue therapy [8]. The ISBI guideline recommends considering reducing metabolism in burn patients to avoid uncontrolled hypercapnia due to hypermetabolism [9]. Both ISBI and DGV guidelines advise against the use of corticosteroids for inhalation injury, and the ISBI, DGV and JSBI advise against prophylactic antibiotics [3,8,9,13]. The various modes of ventilation are not discussed by either guideline except for the JBSI who discusses the potential of high-frequency percussion ventilation [13]. The DGV-guideline, however, recommends returning to early spontaneous breathing [8].

The DGV, JSBI and ISBI guidelines recommend the use of bronchoscopy to assess the severity of a possible inhalational injury and the DGV and ISBI guideline recommend its use to assess potential causes of mechanical ventilation difficulties, such as the presence of bronchial casts [8,9,13]. At the same time, the DGV-guideline advises against endotracheal intubation to facilitate bronchoscopy and against the use of bronchoscopy to remove sooth from the airways [8].

### 3.2. Fluid Resuscitation

All guidelines recommend early and sufficient fluid resuscitation as crucial measure to prevent and treat burn shock, as summarized in Table A3. The EBA, ABA, DGV and ISBI guidelines recommend initiating fluid resuscitation in burns ≥20% TBSA, while the UK, JSBI, IAEM and MSF guidelines recommends a lower threshold of 15% ≥TBSA [4,7,8,9,12,13,14,16]. The EBA and ISBI guidelines recommend calculating the initial fluid infusion rate based on a total fluid resuscitation volume of 2 to 4 mL/kg/TBSA, the IEAM guideline recommends 4 mL/kg/TBSA, the DGV and JSBI guidelines recommend 2 or 4 mL/kg/TBSA and the ABA and MSF guidelines recommend 2 mL/kg/TBSA [4,8,9,12,13,14,16]. The DGV and ISBI guidelines recommend against the use of fluid bolus to improve urinary output and recommend careful titration of the infusion rate, while the MSF recommends an initial fluid bolus of 20 mL/kg in the first hours [8,9,16]. The EBA, ABA, DGV and ISBI guidelines recommend using isotonic crystalloid solutions and the EBA, DGV, JSBI, MSF and ABA guidelines specify using a balanced solution [4,8,9,12,13,14,16]. Furthermore, due to potential concerns regarding increased oxygen consumption by the liver during lactate metabolism and the risk of rebound alkalosis following lactate administration [8], the DGV advises against the use of Ringer’s lactate [19]. The ABA guideline recommends considering albumin administration between 12 and 24 h after burn trauma as an adjunct to improve urinary output, reduce total resuscitation or as a rescue strategy [12]. In contrast, EBA and DGV recommend using albumin only as a rescue when resuscitation goals are not reached with crystalloids only [4,8,12]. The ABA guidelines advise against the use of fresh frozen plasma during resuscitation outside of research protocols [12]. The JSBI guideline gives a weak recommendation for the use of fresh frozen plasma or hypertonic lactate solution and suggests replacing a portion of the resuscitation fluid with hydroxyethyl starch [13]. This recommendation conflicts with other burn guidelines, general intensive care guidelines and regulatory authorities (e.g., FDA and EMA), all of which advise against its use due to safety concerns [4,8,12,20,21].

None of the guidelines recommend the routine use of norepinephrine as a vasopressor during initial burn resuscitation. The EBA guideline advice using norepinephrine only in cases of life-threatening hypotension despite adequate resuscitation [4]. Similarly, the DGV guideline recommends using noradrenaline and the MSF guidelines recommends dopamine or epinephrine in case of persistent hypotension despite resuscitation with adequate volumes of crystalloids and albumin [8,16]. The ABA guideline does not give a recommendation for the use norepinephrine or vasopressin analogues due to lack of evidence [12].

All guidelines recommend using urinary output as primary endpoint for volume resuscitation and for titrating infusion rate, although they specify slightly different target values and ranges. While the ABA and IAEM guidelines recommend a target range of 0.5–1.0 mL/kg/h, the MSF guideline specifies a target value of 1–2 mL/kg/h, the EBA guideline specifies a target value of 0.5 mL/kg/h and the DGV and ISBI guidelines a target range of 0.3–0.5 mL/kg/h [4,8,9,12,13,14,16]. The ISBI suggests that a lower urinary output (<0.5 mL/kg/h) may be acceptable during the first three hours after severe burn trauma [9]. In addition to urine urinary output as the primary endpoint for volume resuscitation, the EBA recommends combining it with mean arterial pressure (>65 mmHg) and lactate (<2 mmol/L) or base excess [4]. The DGV recommends combining urinary output with lactate (<2 mmol/L) or base excess (>−2), heart rate (<110/min), ITBI (<600–800 mL/m^2^), CI (>2.2–3 L/min·m^2^) or ScvO_2_ (>70%) [8]. The MSF guideline recommends combining it with systolic arterial pressure without specifying a target [16]. The EBA guideline is the only one recommending de-escalation of the resuscitation strategy after the first 24 h when clinically feasible [4].

Regarding advanced hemodynamic monitoring, the ABA guideline recommends against using variables derived from transpulmonary thermodilution (such as cardiac index, intrathoracic blood volume index, global end-diastolic volume index, or extravascular lung water index), as these variables may be associated with over infusion. The guideline gives no recommendations for other dynamic parameters, such as stroke volume variation (SVV) or pulse pressure variation (PPV) [12]. The EBA guideline recommends advanced hemodynamic monitoring only in patients who do not respond adequately to volume resuscitation or in complex situations, such as patients with significant comorbidity or trauma. In addition, the EBA guideline recommends the use of dynamic parameters (SVV, PPV or the passive leg raising maneuver) over static parameters (central venous pressure or wedge pressure (PAOP) [4]. In contrast, the DGV guideline recommends using advanced hemodynamic monitoring to potentially detect fluid overload, as excessive or normal values (e.g., ITBVI) may indicate over infusion. In addition, the DGV guideline recommends using advanced hemodynamic monitoring in patients receiving noradrenaline [8]. The JSBI guideline recommends that transpulmonary thermodilution and arterial pulse contour analysis can be used to titrate infusion rate [13].

### 3.3. Pain Management

Burn pain can be severe and pain management is often complex [22]. Burn pain can be classified into acute, procedural, or chronic pain, as well as nociceptive and neuropathic pain [22]. All guidelines provide recommendations for the treatment of acute or nociceptive pain; they are summarized in Table A4. Recommended analgesics regiments include a combination of paracetamol, NSAIDs or metamizole and an opioid [4,5,8,9]. However, none of the guidelines specify most appropriate opioid for use in burn patients. The ABA guideline recommends selecting opioids based on the patient’s physiological status and the physician’s experience [5]. The DGV guideline recommends using gabapentin or pregabalin, amitriptyline and dexmedetomidine or clonidine as adjuvants in the management of acute pain. The ABA guideline recommends dexmedetomidine or clonidine and (es)ketamine [5], while the ISBI guideline recommends clonidine or dexmedetomidine [9]. Most guidelines recommend using an individualized pain management plan based on a locally available burn pain protocol which includes pain assessment tools such as VAS, CCPOT or BPS [4,5,8,9,13,14,16]. Risk factors for neuropathic pain include advanced age, major burn injuries, long hospital stays, alcohol abuse and smoking [23]. The ABA, ISBI and DGV guidelines recommend the additional use of gabapentin or pregabalin for managing neuropathic pain in these patients [5,8,9] and the JSBI guideline recommends the addition of amitriptyline or carbamazepine [13]. Most guidelines recommend additional non-pharmacological interventions such as hypnosis, distraction, relaxation exercises, cognitive behavioral therapy, virtual reality, hypnotherapy and music therapy [4,5,8,9,13]. However, the guidelines do not specify timing, implementation or the most effective therapy.

### 3.4. Procedural Sedation

The recommendations for procedural sedation are summarized in Table A5. The DGV and ISBI guidelines recommend appropriate analgosedation when burn patients must undergo a procedure. While the ISBI guideline recommends avoiding benzodiazepines, the DGV guideline recommends administering a combination of midazolam and esketamine in patients with hemodynamic instability [8]. Upon completion of the weaning process and in anticipation of extubation, the DGV guideline recommends changing the sedation to propofol. Additionally, the ABA, DGV and ISBI guidelines recommend the use of dexmedetomidine, with the ABA guideline recommending dexmedetomidine as the sedative of first choice. The guidelines recommend the use of a validated sedation score (e.g., RASS or SAS score) to monitor the depth of sedation and to establish daily target scores. The DGV guideline further advises daily wake-up calls, especially when using midazolam.

The ABA recommends the use of opioids for analgosedation. Both the ABA and the ISBI suggest considering the addition of (es)ketamine for sedation during procedures [5,9]. In addition, non-pharmacological therapies play a key role [5,8,9]. The DGV guideline recommends the addition of intravenous lidocaine for procedural sedation [8].

## 4. Discussion

Optimal management of burn patients, particularly in the acute phase, requires a nuanced, multidisciplinary approach. Current international and national guidelines—including those from ISBI, DGV, EAST, ABA, JSBI, IAEM, MSF and EBA—provide essential guidance for clinical decision making. But critical gaps exist in their specificity and applicability, especially for clinicians without specialized burn care expertise.

Airway management remains a complex and dynamic challenge in burn care. Although existing guidelines provide burn-specific intubation triggers, they often lack clear decision-making algorithms. Advanced airway techniques such as nasopharyngoscopy and videolaryngoscopy [24] receive little attention, despite their potential to play a vital role in assessing airway patency, risk for airway obstruction and guiding safe intubation strategies. Future guidelines should incorporate these techniques into flowcharts, as they enable direct visualization of the nasopharynx, epiglottis and vocal cords providing valuable insight in the progression of thermal injury to the oropharynx [24]. Videolaryngoscopy is a useful tool for difficult airways and can be used as the primary intubation strategy, giving its association with higher first-attempt success [25].

With regard to mechanical ventilation modes, the current guidelines recommend elements of the current state of the art in mechanical ventilation for ARDS. There are no recommendations for airway pressure relief ventilation (APRV) or high-frequency percussion ventilation (HPFV), although these are commonly used in the US [26]. To date, there is no evidence that burn patients require different mechanical ventilation strategies than non-burn patients [24], and the most recent consensus statement classified HFPV as inappropriate for burn patients [27]. Future guidelines for burn patients should include a more detailed approach to lung-protective ventilation and also include recommendations on the potential benefits of using APRV and HFPV.

Fluid resuscitation continues to be a cornerstone of early burn care in the first 24 to 48 h after severe burn trauma. While there is consensus among guidelines regarding the use of isotonic crystalloids, there are divergent recommendations on the use of adjuncts such as albumin, vasopressors and advanced hemodynamic monitoring reflecting gaps in evidence. There is also consensus in the guidelines regarding urinary output as endpoint; however, it would be useful to examine whether newer, more sophisticated approaches could complement urinary output, with its limitations as the sole endpoint. Concepts such as fluid reactivity and fluid tolerance could serve as a starting point for recommendations in future guidelines [28,29,30]. In addition, future guidelines should also include recommendations for the integration of multiple hemodynamic parameters to guide volume resuscitation. Furthermore, the role of norepinephrine, which is well established in general critical care, remains insufficiently defined in the context of burn shock [31].

Similarly, the precise role of albumin in burn resuscitation remains under debate. Although recent studies have shown that adjunctive albumin can reduce the total volume of resuscitation [32,33,34], consensus regarding its optimal dose, timing and specific indications in burn patients remain lacking. These aspects are currently being investigated in the multicenter, randomized, controlled ABRUPT-II trial (NCT04356859). Frozen fresh plasma has also shown promising results in animal studies, where it reduced endothelial dysfunction and the total resuscitation volume required in burn shock [35]. However, clinical evidence supporting its efficacy in clinical practice is still lacking [12,34].

The management of acute burn pain has evolved into a multimodal approach, and the guidelines recommend integrating both pharmacologic and non-pharmacologic approaches. In contrast, the treatment of chronic and neuropathic pain remains underrepresented in the guidelines despite its high prevalence and significant impact on patients’ quality of life. Moreover, there is a need for improved guidelines regarding procedural pain, analgesic and sedative drug selection and long-term pain management strategies. Among the reviewed guidelines, only the ISBI guideline addresses this issue and recommends primarily focusing on non-opioid treatments [9].

The care of burn patients is demanding and requires considerable resources in terms of infrastructure and expertise. These requirements can pose significant challenges in resource-limited settings or circumstances, such as armed conflicts or mass casualty incidents [36]. Importantly, such scenarios can also occur in resource-rich countries, underscoring the need for inclusion in future guidelines. Currently, the guidelines lack specific recommendations for the treatment of critical burns in these contexts. Future guidelines should close this gap by including recommendations for burn patients in resource-limited settings.

## 5. Conclusions

In summary, the current guidelines provide useful recommendations for the initial management of vital signs. While most recommendations are consistent across guidelines, some discrepancies reflect gaps in evidence. Future guidelines should include specific recommendations on the use of advanced airway management techniques, fluid resuscitation endpoints and strategies for managing chronic pain.

## Data Availability

The original contributions presented in this study are included in the article Further inquiries can be directed to the corresponding author.

## Abbreviations

The following abbreviations are used in this manuscript:

ABA	American Burn Association
BBA	British Burn Association
BE	Base excess
BPS	Behavioral pain scale
BSPAS	Burn specific pain anxiety score
CBT	Cognitive behavioral therapy
CCPOT	Critical care pain observation tool
CI	Cardiac index
CO	Carbon monoxide
CVP	Central venous pressure
DGV	German Society for Bun Medicine/Deutsche Gesellschaft für Verbrennungsmedizin
EAST	Eastern Association for the Surgery of Trauma
EBA	European Burn Association
EMA	European Medicines Agency
EVLWI	Extra-vascular lung water index
FDA	U.S. Food and Drug Administration
FFP	Fresh frozen plasma
GEDVI	Global end-diastolic volume index
HES	Hydroxyethyl starch
HLS	Hypertonic lactated solution
IAEM	Irish Association of Emergency Medicine
ITBVI	Intrathoracic blood volume index
ISBI	International Society for Burn Injuries
JSBI	Japanese Society for Burn Injuries
MSF	Medicines sans Frontieres
NSAID	Non-steroidal anti-inflammatory drug
PAOP	Pulmonary artery occlusion pressure
PEEP	Positive end-expiratory pressure
PLR	Passive leg raise
PPV	Pulse pressure variation
RASS	Richmond agitation-sedation scale
SAS	Sedation-agitation scale
ScvO2	Central venous oxygen saturation
SVV	Stroke volume variation
TBSA	Total body surface area
VAS	Visual analogue scale
vv-ECMO	Veno-venous extracorporeal membrane oxygenation

## Appendix A

**Table A1:** Airway Management.

	EBA	ABA	DGV	ISBI	EAST	JSBI	IAEM	MSF
Intubation trigger	NA	NA	Acute respiratory failure, lack of protective reflexes, risk of loss of patency in case of severe inhalation trauma and thermal damage to the mucous membrane of the mouth and throat.	Acute respiratory failure, systemic inhalation injury, thermal injury to the face, mouth or oropharynx threatening airway patency.	Airway obstruction, severe cognitive impairment (GCS < 9), large burns (>40%), prolonged anticipated time to definitive care, risk of losing airway patency based on moderate-to-severe facial or oropharyngeal burns or airway injury on endoscopy.	NA	Acute respiratory failure, possible airway involvement: stridor, hoarseness, edema/erythema on oropharyngeal inspection, circumferential burns of neck.	NA
Monitoring need for intubation	NA	NA	Signs of threatened patency: inspiratory stridor, multi-trauma and circular torso burns), circular burns of the neck. Large burns are no indication for ventilation without other intubation triggers.	Continues monitoring and frequent assessment are essential. Signs of threatened patency: burning buccal cavity (blistering of mucosal membrane), hoarseness or stridor. Factors of influence for the trigger: expertise and facilities of hospital and clinical insight of physician in charge.	NA	Two opinions: prophylactic early intubation or in case of symptoms of upper airway obstruction after careful monitoring. Decision based on expertise of medical staff and capacity facility.	Any patient with signs or airway or inhalation injury should be handled by the most experienced clinician.	NA
Strategy for intubation	NA	NA	NA	Most experienced clinician should perform the airway maneuver.	NA	NA	Most experienced clinician should perform the airway maneuver.	NA
Preventive strategy	NA	NA	NA	Semi-upright position with moderate elevation of the head and trunk. Oxygen therapy to maintain adequate arterial saturation. Oral suctioning to prevent build-up of debris and secretions.	NA	NA	NA	NA

EBA: European Burn Association, ABA: American Burn Association, DGV: Deutsche Gesellschaft für Verbrennungsmedizin, ISBI: International Society for Burns Injuries, EAST: Eastern Association for Surgery of Trauma, JSBI: Japanese Society of Burn Injuries, IAEM: Irish Association of Emergency Medicine, MSF: Medicine Sans Frontieres, NA: not available within the documents of the respective organization.

**Table A2:** Mechanical ventilation and respiratory management.

	EBA	ABA	DGV	ISBI	BBA	JSBI	IAEM	MSF
Mode of ventilation	NA	NA	Early spontaneous breathing.	NA	NA	Opinions for: conventional or high-frequency percussion ventilation	NA	NA
Tidal Volumes	NA	NA	6–8 mL/kg	NA	NA		NA	NA
PEEP-strategy	NA	NA	≥5 cm H_2_O	NA	NA		NA	NA
Goals	NA	NA	End-inspiratory pressure ≤30 cm H_2_O. Driving pressure ≤15 cm H_2_O. Permissive hypercapnia if needed. Limit the ventilation time as much as possible.	Lung protective ventilation, with the lowest tidal volume and airways pressures as possible based on CO_2_ clearance.	NA	Opinions for: low-tidal ventilation	NA	NA
Rescue therapy	NA	NA	Prone position VA-ECMO	NA	NA		NA	NA
Bronchoscopy strategy	NA	NA	In case of suspicion for inhalation trauma and pulmonary impairment in mechanical ventilated patients, bronchoscopy should be performed. The guidelines advised against bronchoscopy for a non-mechanical ventilated patients and against the removal of sooth.	Diagnosis inhalation injury in the presence of damaged mucosa below the larynx.	NA	Diagnosis of inhalation injury	NA	NA
Corticosteroids Antibiotics	Routine use of prophylactic systemic antibiotics is not advised.	NA	Routine use of prophylactic systemic antibiotics or corticosteroids is not advised.	Routine use of prophylactic systemic antibiotics or corticosteroids is not advised.	NA	Routine use of prophylactic systemic antibiotics is not advised	NA	NA
Carbon monoxide intoxication/cyanide intoxication	NA	NA	CO: FiO_2_ 100%/hydroxocobalamin	CO: High flow oxygen for at least 6 h (8–15 L non-rebreathing mask). Insufficient evidence for hyperbaric oxygen therapy Cyanide: Immediate hydroxocobalamin administration.	NA		CO: FiO_2_ 100% Cyanide: FiO_2_ 100%, hydroxocobalamin in severe lactate acidosis, comatose or cardiovascular compromise, Sodium thiosulphate as adjuvant.	NA
Weaning	NA	NA	The possibility of late inhalation trauma should not delay weaning	NA	NA		NA	NA

EBA: European Burn Association, ABA: American Burn Association, DGV: Deutsche Gesellschaft für Verbrennungsmedizin, ISBI: International Society for Burns Injuries, JSBI: Japanese Society of Burn Injuries, IAEM: Irish Association of Emergency Medicine, MSF: Medicine Sans Frontieres, NA: not available within the documents of the respective organization, VA-ECMO: venous-arterial extracorporeal membrane oxygenation, CO: carbon monoxide.

**Table A3:** Fluid and resuscitation strategy.

	EBA	ABA	DGV	ISBI	BBA	JSBI	IAEM	MSF
Threshold starting resuscitation	>20% TBSA	>20% TBSA	>20% TBSA	>20% TBSA	>15% TBSA	>15% TBSA	>15% TBSA	>15% TBSA
Calculate initial fluid rate	Between 2 and 4 mL/kg/TBSA, half within first 8 h.	2 mL/kg/TBSA	2- or 4 mL/kg/TBSA, half within first 8 h. Avoid bolus administration.	2–4 mL/kg/TBSA, alertness to over-resuscitation. Avoid bolus administration.	NA	2- or 4 mL/kg/TBSA	4 mL/kg/TBSA Use fluid bolus to stabilize prior to continuous infusion	20 mL/kg first hour 2 mL/kg/TBSA
Type of fluid	Balanced crystalloid solution Avoid standard colloids, consider albumin as rescue, not before 8 h.	Balanced crystalloid solution. Consider albumin after 12 to 24 h, as a rescue when resuscitation is failing despite adequate crystalloid suppletion.	Balanced solution, for example, Ringer’s acetate but not Ringer’s lactate. Consider albumin as a rescue in case of failing resuscitation	Salt containing fluid.	NA	Ringer’s lactate Weak recommendation for FFP and HLS. Suggested to replace portion of crystalloid volume with HES.	NA	Ringer’s lactate
Vasoactive medication	Life threatening hypotension despite adequate resuscitation. Inotropic agents should only be used in case of reduced cardiac function.	No recommendation can be given regarding noradrenalin, or vasopressin analogues.	If resuscitation using crystalloid and albumin does not achieve stabilization, noradrenaline should be preferred.	NA	NA	NA	NA	Persistent oliguria despite adequate resuscitation: dopamine or epinephrine
Advanced hemodynamic monitoring	In severe burn patients not responding as expected or complex situation (e.g., trauma or comorbidity). Use dynamic measurements (e.g., SVV, PPV or PLR) Do not use static measurements (e.g., CVP, PAOP)	Do not recommend the use of transpulmonary thermodilution-derived variables to reduce total resuscitation volume or edema-related complications (CI, ITBVI, EVLW, GEDVI) No recommendation on SVV or PPV.	Advanced hemodynamic monitoring can be used to indicate over-infusion, as (supra)normal value suggest adequate volume status. Advanced monitoring should always be used when starting a vasopressor.	NA	NA	Transpulmonary thermodilution or arterial pulse contour analysis can be used to titrate infusion rate.	NA	NA
Strategy of tapering fluids	De-escalation of fluid therapy after the first 24 h.	NA	Every 2–3 h adjustments should be made. Diuresis > 1mL/kg/h represent over-resuscitation	NA	NA	NA	NA	NA
End point of resuscitation	Choice of endpoint or monitoring should be based on the expertise of the physician and patient characteristics, using: - MAP around 65 mmHg, with individualized goals. - Lactate or BE, lactate > 2 mmol/L suggest hypoperfusion. - Urinary production > 0.5 mL/kg/h, most useful in case in resource-limited settings. - Use SvO_2_ to help assess tissue perfusion.	Urinary output 0.5–1 mL/kg/h	Therapy should not be guided by focusing on a single target but on the overall clinical picture, using: - Urinary output 0.3–0.5 mL/kg/h - Lactate < 2 mmol/L or BE > −2 - Heart rate < 110 min - ITBVI < 600–800 mL/m^2^ - CI > 2.2–3 L/min × m^2^ - ScvO_2_ > 70% - MAP > 65 mmHg	Urinary output 0.3–0.5 mL/kg/h First three hours anuria possibly does not respond to fluid additional fluid administration.	NA	Advice for respiratory and circulatory monitoring, including urinary output.	Urinary output 0.5–1 mL/kg/h	Appropriate systolic arterial blood pressure Urinary output 1–2 mL/kg/h

**Table A4:** Pain Management.

	EBA	ABA	DGV	ISBI	BBA	JSBI	IAEM	MSF
Concept	Protocol based, multi-disciplinary approach. Continuous and accurate assessment of pain and response to therapy. Aggressive pain management	Pain assessments must be performed regularly and be protocolized. Minimize opioids as much as possible.	Standardized assessment every 8 h.	Routine use of scoring systems during all phases of care. Protocolized pain management strategy.	NA	Ensure adequate analgesia, titrate to the minimum dose. NSAIDS can be considers as alternatives.	Ensure adequate analgesia, document pain score, and reassess post administration.	Regular assessment
Acute/background pain	Individualized management plan. WHO Pain Ladder	Combine opioids with nonopioids (acetaminophen, consider NSAID) and nonpharmacologic interventions. Individualize opioid choice on patient’s physiology and expertise physician. In refractory pain, without neuropathic component, perform trial of gabapentin or pregabalin.	Multimodal concept consisting of analgesic, adjuvants and nonpharmacologic methods: - Paracetamol - Metamizole or NSAID - Opioids	Individualized multimodal approach should be considered using: - Acetaminophen. - NSAID. - Opioids - Nonpharmacological interventions.	NA	Intravenous opioids as first choice for analgesia in combination with	Early administration of intravenous opioids	Moderate: paracetamol and tramadol Moderate to severe: paracetamol and sustained release morphine
Chronic pain	NA	NA	NA	WHO pain ladder, emphasis on nonopioids, stepwise addition of other agents.	NA	NA	NA	Self-evaluation and paracetamol and/or tramadol.
Procedural pain	NA	Use opioids as the basis. Ketamine can be considered. Nonpharmacologic interventions.	Intravenous lidocaine.	Ketamine can be considered for procedural pain.	NA	NA	NA	Mild to moderate: tramadol, addition of morphine when needed Moderate to severe: immediate release morphine oral or subcutaneous As last resort: ketamine
Adjuncts	Consider anxiolysis.	Dexmedetomidine and clonidine, particular in withdrawal of prominent anxiety symptoms. Ketamine to reduce opioids, particularly in postoperative period.	Antiemetics and laxatives. Gabapentin or pregabalin can be used as a modality, however not as pain preventive strategy Amitriptyline can be used as a modality. Clonidine or dexmedetomidine.	Dexmedetomidine can be used to reduce opioid requirements	NA	NA	NA	NA
Neuropathic pain	NA	Add gabapentin or pregabalin.	Add gabapentin or pregabalin.	Add gabapentin or pregabalin.	NA	NA	NA	Amitriptyline or carbamazepine
Regional anesthesia	NA	Yes, reduce pain, higher satisfaction and reducing opioid use.	Yes, peripheral nerve blockade (ideally with catheter) and epidural analgesia can be considered.	NA	NA	NA	NA	NA
Nonpharmacologic interventions	Active hypnosis, rapid induction analgesia, distraction relaxation.	Non-pharmacologic technique should be used in every patient. (e.g., CBT, hypnosis and VR)	Coping strategies, hypnotherapy, CBT, VR	Manage emotional factors. Important to add nonpharmacologic intervention (e.g., education, distraction, relaxation, music therapy or hypnosis)	NA	Physical/occupation therapy, psychological counseling and other alternative therapies.	NA	NA
Audit Tools	VAS	BSPAS/CCPOT	NRS/VAS/BPS	BPS/CCPOT/NRS/VAS	NA	NRS/VAS/BPS/CPOT	Use a pain score.	SVS

EBA: European Burn Association, ABA: American Burn Association, DGV: Deutsche Gesellschaft für Verbrennungsmedizin, ISBI: International Society for Burns Injuries, JSBI: Japanese Society of Burn Injuries, IAEM: Irish Association of Emergency Medicine, MSF: Medicine Sans Frontieres, NA: not available within the documents of the respective organization, CBT: cognitive behavioral therapy, VR: virtual reality, BSPAS: Burn Specific Pain Anxiety Scale, CCPOT: Critical Care Pain Observation Tool, BPS: behavioral pain score, NRS: numeric rating scale, VAS: Visual Analog Scale, SVS: simple verbal scale.

**Table A5:** Sedation management.

	EBA	ABA	DGV	ISBI	BBA	JSBI	IAEM	MSF
Approach	NA	NA	Analgosedation. Sedation should not be used on indication (e.g., anxiety or agitation)	Analgosedation. Light sedation (patient arousable and able to purposefully follow simple commands)	NA		NA	NA
Nonpharmacologic measures	NA	NA	Reduction in light, noise and restriction to necessary measures at night	Optimizing the environment, early mobilization, diversion therapy and frequent reorientation.	NA		NA	NA
Monitoring sedation depth	NA	NA	A goal should be defined using the BPS or RASS score. Daily awake-up call, especially when using midazolam sedation.	Use RASS or SAS to monitor depth of sedation.	NA		NA	NA
Agents of sedation	NA	Dexmedetomidine as fist line treatment in intubated patients.	There is no clearly superior analgosedation strategy. The combination of esketamine and midazolam is the most hemodynamic stable choice (midazolam in lowest possible dose). When extubation is planned, while using midazolam, timely transition to propofol is needed. Dexmedetomidine can be added.	Nonbenzodiazepine medications are preferred over benzodiazepine (e.g., propofol and dexmedetomidine)	NA	Nonbenzodiazepine medications are preferred over benzodiazepine. Consider ketamine.	NA	NA

EBA: European Burn Association, ABA: American Burn Association, DGV: Deutsche Gesellschaft für Verbrennungsmedizin, ISBI: International Society for Burns Injuries, JSBI: Japanese Society of Burn Injuries, IAEM: Irish Association of Emergency Medicine, MSF: Medicine Sans Frontieres, NA: not available within the documents of the respective organization, RASS: Richmond Agitation-Sedation Scale, SAS: Sedation-Agitation Scale.
